# Trends and age-period-cohort effects on incidence and mortality of asthma in Sichuan Province, China, 1990–2019

**DOI:** 10.1186/s12890-022-02059-y

**Published:** 2022-08-03

**Authors:** Yu Luo, Mu Wang, Yumei Tian

**Affiliations:** 1 Department of Obstetrics Nursing, West China Second University Hospital, Sichuan University, Chengdu, China; 2 West China School of Nursing, Sichuan University, Chengdu, 610000 Sichuan China; 3 Key Laboratory of Birth Defects and Related Diseases of Women and Children (Sichuan University), Ministry of Education, Chengdu, 610000 Sichuan China; 4 Outpatient Department, Mianyang Central Hospital, School of Medicine, University of Electronic Science and Technology of China, Mianyang, 621000 Sichuan China

**Keywords:** Asthma, Age-period-cohort effect, Incidence, Mortality, Sichuan Province

## Abstract

**Background:**

The provinces in western China have undergone rapid urbanization and industrialization, particularly since the Chinese government launched the Great Western Development Strategy in 2000. We examined the time trends and contributions of age, period, and cohort effects to asthma incidence and mortality in Sichuan Province, a populous province in western China, from 1990 to 2019.

**Methods:**

The data of Sichuan Province from 1990 to 2019 were extracted from the Global Burden of Disease study 2019. Trends and average annual percentage change were estimated using joinpoint regression. Age, period, and cohort effects were estimated using an age-period-cohort model with the intrinsic estimator method.

**Results:**

In the latest period (2015–2019), the highest incidence of asthma was 2004.49/100,000 in children aged < 5 years, and the highest mortality rate was 22.04/100,000 for elderly people aged > 80 years. Age-standardized rates generally remained stable (95% confidence interval [CI]  − 0.21, 0.11) for incidence and declined by 4.74% (95% CI − 5.09, − 4.39) for mortality over the last 30 years. After controlling for other effects, the age effect on asthma showed that the incidence rate ratio (RR) was highest in the < 5 years age group, and the mortality RR was highest in the > 80 years age group. The period effect on incidence and mortality decreased from 1990 to 2019, respectively. A cohort effect was found the incidence RR increased slowly from the early birth cohorts to the later birth cohorts, especially after the 2005 birth cohort, whereas the mortality RR continued to decline.

**Conclusions:**

There was a significant effect of older age on the asthma mortality rate over the last 30 years, and the incidence rate in children aged < 5 years increased. The relative risk of asthma incidence in the later birth cohorts increased. Effective preventive measures and public health policies should be to protect children and elderly people from potentially harmful chronic diseases.

## Background

In 2019, chronic respiratory diseases were the third most frequent cause of global mortality and the ninth most frequent cause of disability-adjusted life years (DALY); of such diseases, asthma is one of the most common [1, 2]. Asthma affects all age groups, but particularly children; in 2019, asthma affected an estimated 262 million people and caused 461,000 deaths globally [2, 3]. In China alone, according to the Global Burden of Disease (GBD) study 2019, there were 3,761,277 new cases and 24,750 deaths from asthma in 2019 [4]. Some reports have suggested that there are currently many undiagnosed and undertreated cases of asthma in China [5]. Similar to many chronic non-communicable diseases, asthma is not only a complex genetic disorder, but is also substantially affected by environmental, occupational, lifestyle, and behavioral factors [6–8]. Many recent studies have shown that asthma prevalence in developed regions is higher than that in developing regions, and is also higher in urban residents than in rural residents [2, 9]. Areas in which there has been a rapid rise in asthma incidence, such as Africa and Eastern Mediterranean, are generally those characterized by rapid social and economic development, whereas upper-middle-income and high-income areas have been relatively stable [9, 10]. Since the Chinese government launched the Great Western Development Strategy in 2000, the western region of China has undergone rapid urbanization and industrialization. This may have contributed to asthma prevalence and the resulting substantial burden of disease [11–13]. Huang and his colleagues assessed trends in asthma mortality in China from 2000 to 2019, and showed that age, period, and population birth cohorts may contribute to changes in asthma mortality [14]. At present, the trends in asthma incidence and mortality in the western provinces of China are unclear. To support the implementation of the Chronic Respiratory Diseases Prevention and Control Campaign in Healthy China Action 2019–2030, we systematically analyzed the changes in asthma in Sichuan Province, a populous province in western China [15].

Sichuan province is the most populous province (with more than 80 million residents) of the 12 Great Western Development Strategy policy regions. Because of its large size (486,000 km), multiethnic composition, and unbalanced economic development, Sichuan is considered a typical Chinese province [16]. In this study, we examined asthma incidence and mortality in Sichuan from 1990 to 2019 using age-period-cohort (APC) analysis. APC analysis has been widely used to address important questions related to social change, etiology of disease, aging, and population processes and dynamics and to demonstrate the effects of age, period, and cohort factors [17]. Age effects represent changes in disease incidence and mortality because of aging processes, which arise from physiological changes, accumulation of social experience, social role or status changes, or a combination of these. Period effects subsume a complex set of historical events and environmental factors that affect all age groups simultaneously. Cohort effects refer to the difference between different birth cohort groups. Some studies have shown that social and environmental factors may drive period and cohort effects; for example, policies or economic changes can affect air pollution levels, tobacco use, and asthma awareness and diagnosis [18, 19]. The aim of this study was to examine time trends and the unique effects of age, period, and cohort, as well as the simultaneous synergistic effects of all three, to provide theoretical support for health policymaking in regions characterized by rapid economic change.

## Methods

### Data source

Data on asthma incidence and mortality rates in Sichuan were obtained from the GBD study 2019. The GBD study provided a comprehensive estimation of 369 diseases and injuries by age and sex in 204 countries and territories from 1990 to 2019 [20]. The GBD study synthesized data from a large number of input sources to obtain estimated disease burden indexes, including incidence and mortality. The original data estimated by the GBD study for asthma incidence and mortality in China is briefly summarized. These data were mainly obtained from published cross-sectional studies, cohort studies, and multicenter epidemiological studies in China [21–23], and from nationally representative institutions such as the Cause of Death Reporting System of the Chinese Centers for Disease Control and Prevention, the Disease Surveillance Points, and the Maternal and Child Surveillance System. More detailed information about data input sources can be found on the GBD study website (https://ghdx.healthdata.org/gbd-2019/data-input-sources) and in previous reports [24–28]. This project received waiver for ethical approval from Sichuan University (2022-128).

### Asthma incidence and mortality analysis

A Bayesian meta-regression model generated in DisMod-MR 2.1 was used to uniformly estimate asthma incidence and mortality by the GBD study, the standardized methods of which have been extensively reported elsewhere [29, 30]. The incidence and mortality rate was calculated for each age group (e.g., 0–4, 5–9 years) up to ≥ 80 years. The age-standardized incidence rate (ASIR) and age-standardized mortality rate (ASMR) for asthma were calculated using direct standardization according to the GBD world population standard [31]. These data including asthma incidence and mortality by age and year, ASIR and ASMR were used to perform a joinpoint regression and APC analysis.

### Joinpoint Poisson regression

Identification of temporal trend change is an important issue in the analysis of disease mortality and incidence rate. Joinpoint regression establishes segmental regression based on temporal characteristics of disease distribution. The logarithmic linear model was selected and the rates were transformed logarithmically. All possible connection points of the interval piecewise function were established using a grid search method. To comprehensively evaluate the total average trend in incidence and mortality rate, the average annual percentage changes (AAPC) and corresponding 95% confidence intervals (CI) were assessed using joinpoint regression analysis [32]. The Monte Carlo permutation test method was used to optimize the model, and the Bonferroni correction was used to maintain the overall asymptotic significance level [33]. The analysis was performed using “joinpoint” software developed by the Division of Cancer Control and Population Sciences of the US National Cancer Institute [34].

### APC analysis

The APC model is based on the Poisson distribution and was used to ascertain temporal trends in asthma by age, period, and cohort, as well as the trends after adjusting for age, period, and cohort [35]. The asthma incidence and mortality rates between 1990 and 2019 were separately modeled using the Poisson log-linear model. The model can be expressed as:

$${\mathrm{logr}}_{\mathrm{ij}}=\mu +{\mathrm{\alpha }}_{\mathrm{i}}+{\upbeta }_{\mathrm{j}}+{\upgamma }_{\mathrm{k}},\mathrm{i}=1,\dots ,\mathrm{a}.\mathrm{ j}=1,\dots ,\mathrm{p}$$

where r_ij_ denotes the expected incidence or mortality of asthma for the i-th age group and the j-th examination year; μ denotes the overall population mean; α_i_ denotes the effect of the i-th age group; βj denotes the effect of the j-th examination year; and γ_k_ denotes the effect of the k-th cohort. We used the intrinsic estimator method based on the intention-to-collapse method to estimate the age, period, and cohort effects, which provide unbiased and relatively efficient estimation results. The intention-to-collapse method required the continuous periods to be collapsed into one period to ensure the age groups had the same time span. As the age groups were grouped 5 years apart (0–4, 5–9, …, 75–79, ≥ 80 years), five continuous periods (1990–1994, 1995–1999, …, 2015–2019) were also collapsed into one period to ensure the same time span as the age groups. Because the intention-to-collapse method does not change the coding of the age and period effects, for intuitive explanation, we calculated the rate ratio (RR) as the exponential values of the regression coefficients (exp(coef.) = e^coef.^) [36]. This measure describes the incidence and mortality relative risk of asthma for a particular age, period, or cohort compared with the overall average rate. The analysis was performed using the apc package in R language (version 4.1.2).

## Results

### Asthma incidence and mortality in Sichuan Province

**Fig. 1:**
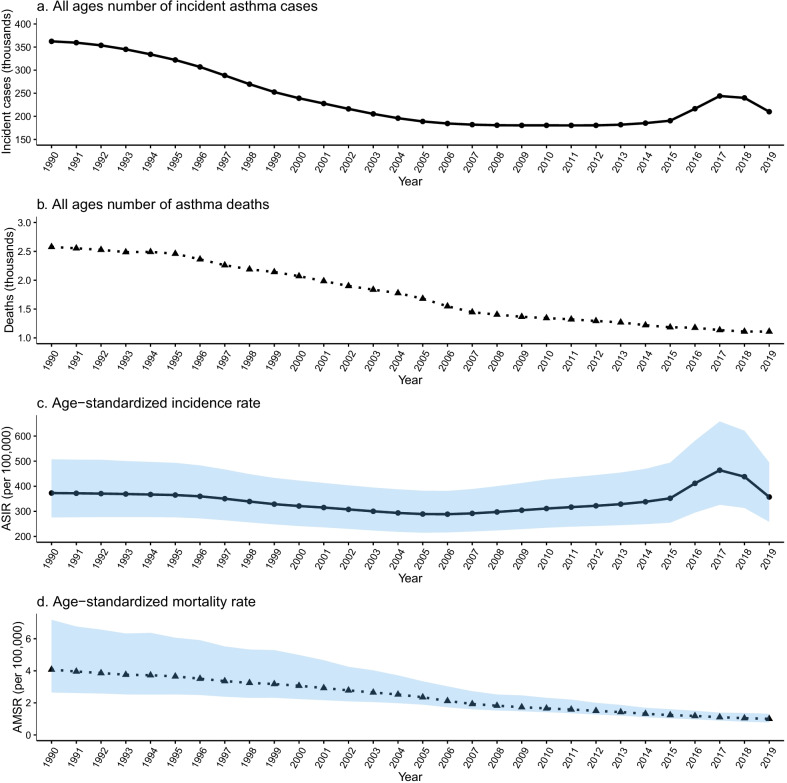
Time trends in asthma ASIR (**a**) and AMSR (**b**) in Sichuan Province, 1990–2019. *ASIR* age-standardized incidence rate; *ASMR* age-standardized mortality rate; *CI* confidence interval. Shadow = 95% Confidence Interval

Figure 1 shows that the ASIR in Sichuan decreased from 372.84/100,000 in 1990 to 288.56/100,000 in 2006, then gradually increased to 463.89/100,000 in 2017 and finally dropped to 357.18/100,000 in 2019. The ASMR decreased from 4.07/100,000 in 1990 to 1.01/100,000 in 2019. The total number of incident cases and deaths fluctuated similarly to ASIR and ASMR. Table 1 shows the age distributions of asthma incidence and mortality rates. In the latest period (2015–2019), the highest incidence of asthma was 2004.49/100,000 for individuals aged < 5 years, and the lowest incidence rate was 90.61/100,000 for those aged 45–49 years. The mortality rate was highest for those aged > 80 years (22.04/100,000), and the age-specific asthma mortality rate increased with age.

**Table 1:** Age-specific asthma incidence and mortality rates (per 100,000) in Sichuan by 5-year periods 1990–2019

Age group (year)	Incidence						Mortality					
	1990–1994	1995–1999	2000–2004	2005–2009	2010–2014	2015–2019	1990–1994	1995–1999	2000–2004	2005–2009	2010–2014	2015–2019
Under 5	1318.54	1238.27	1064.02	1123.84	1490.94	2004.49	0.89	0.48	0.20	0.09	0.05	0.03
5–9	748.36	680.99	585.07	577.48	586.82	816.64	0.22	0.19	0.11	0.06	0.04	0.03
10–14	401.38	404.89	396.75	329.61	289.54	326.33	0.21	0.19	0.11	0.06	0.05	0.03
15–19	258.19	262.72	256.85	220.37	203.01	211.40	0.21	0.17	0.12	0.08	0.05	0.04
20–24	166.39	164.92	150.55	136.92	133.68	140.45	0.29	0.28	0.20	0.11	0.08	0.06
25–29	126.98	123.60	106.13	93.14	91.34	104.38	0.45	0.47	0.34	0.18	0.13	0.10
30–34	111.96	107.73	89.29	77.28	76.22	92.17	0.58	0.59	0.43	0.24	0.17	0.13
35–39	123.13	117.64	102.40	89.13	87.71	99.28	0.85	0.76	0.58	0.39	0.26	0.22
40–44	126.44	120.97	103.70	89.74	88.05	98.36	1.34	1.16	0.89	0.61	0.45	0.39
45–49	122.94	116.22	94.55	79.33	77.23	90.61	1.64	1.55	1.13	0.75	0.61	0.47
50–54	137.00	128.12	105.50	89.77	86.47	96.14	2.44	2.09	1.63	1.13	0.85	0.69
55–59	168.39	155.85	139.99	121.13	115.93	115.41	3.64	2.88	2.35	1.71	1.28	1.11
60–64	251.91	232.88	211.02	184.09	164.70	149.91	5.94	5.00	3.79	2.76	2.20	1.65
65–69	381.03	355.72	317.21	277.16	234.10	199.33	10.22	8.87	7.23	4.88	3.84	2.81
70–74	385.43	366.01	333.78	295.55	247.90	212.36	20.43	18.19	14.49	9.81	6.63	4.90
75–79	278.35	272.31	263.70	242.29	207.42	188.17	33.41	30.39	26.36	17.67	12.45	9.27
80 plus	298.74	298.15	304.41	304.86	280.14	261.56	66.60	57.74	52.54	41.11	31.08	22.04

### Trends in age-specific incidence and mortality rates using joinpoint regression analysis

Table 2 shows the AAPC of asthma incidence and mortality rates in Sichuan from 1990 to 2019. Age-standardized rates declined by 0.05% (95% CI − 0.21, 0.11) for incidence and 4.74% (95% CI − 5.09, − 4.39) for mortality over the last 30 years. Moreover, ASIR slightly declined by 0.39% (95% CI − 0.67, − 0.11) from 1990 to 1994, further declined by 2.46% (95% CI − 2.57, − 2.34) from 1995 to 2004, significantly rose by 1.65% (95% CI 1.51, 1.79) from 2005 to 2013, further rose by 12.13% (95% CI 10.73, 13.55) from 2014 to 2016, then declined by 11.20% (95% CI − 12.31, − 10.08) from 2017 to 2019. ASMR declined over the same period. From 1990 to 1994, 1995 to 1999, 2000 to 2003, 2004 to 2006, 2007 to 2011, and 2012 to 2019, ASMR decreased by 2.21%, 3.35%, 4.51%, 8.76%, 4.96%, and 5.73%, respectively. In the age-specific incidence rates, slightly increases were observed in age groups < 25 years, and slight decreases were observed in age groups > 25 years from 1990 to 2019. Mortality rates simultaneously declined in all age groups. Compared with the older groups, the younger age groups showed more substantial mortality decline. Overall, the asthma ASIR has remained stable, whereas mortality has decreased, among all age groups over the last three decades.

**Table 2:** Average annual percentage changes in asthma incidence and mortality in Sichuan Province, 1990–2019

Age group (year)	Incidence		Mortality	
	AAPC	95% CI	AAPC	95% CI
ASR	1990 to 2019: − 0.05	(− 0.21, 0.11)	1990 to 2019: − 4.74*	(− 5.09, − 4.39)
	1990 to 1994: − 0.39*	(− 0.67, − 0.11)	1990 to 1994: − 2.21*	(− 2.78, − 1.64)
	1995 to 2004: − 2.46*	(− 2.57, − 2.34)	1995 to 1999: − 3.35*	(− 4.14, − 2.55)
	2005 to 2013:1.65*	(1.51, 1.79)	2000 to 2003: − 4.51*	(− 5.75, − 3.26)
	2014 to 2016:12.13*	(10.73, 13.55)	2004 to 2006: − 8.76*	(− 11.10, − 6.36)
	2017 to 2019: − 11.20*	(− 12.31, − 10.08)	2007 to 2011: − 4.96*	(− 5.74, − 4.17)
			2012 to 2019: − 5.73*	(− 6.06, − 5.40)
Under 5	0.31	(− 0.76, 1.39)	− 11.94*	(− 12.28, − 11.60)
5–9	0.13	(− 0.26, 0.51)	− 8.11*	(− 9.05, − 7.17)
10–14	0.10	(− 0.11, 0.30)	− 7.48*	(− 8.33, − 6.62)
15–19	0.12	(− 0.12, 0.36)	− 6.41*	(− 6.87, − 5.95)
20–24	0.06	(− 0.10, 0.22)	− 5.87*	(− 6.65, − 5.09)
25–29	− 0.02	(− 0.21, 0.18)	− 5.73*	(− 6.71, − 4.73)
30–34	− 0.12	(− 0.44, 0.19)	− 5.66*	(− 6.63, − 4.68)
35–39	− 0.34*	(− 0.48, − 0.21)	− 5.09*	(− 5.50, − 4.68)
40–44	− 0.50*	(− 0.66, − 0.35)	− 4.66*	(− 4.89, − 4.42)
45–49	− 0.61*	(− 0.93, − 0.29)	− 4.89*	(− 5.66, − 4.11)
50–54	− 1.03*	(− 1.39, − 0.67)	− 4.83*	(− 5.22, − 4.45)
55–59	− 1.41*	(− 1.59, − 1.23)	− 4.70*	(− 5.02, − 4.38)
60–64	− 1.88*	(− 1.91, − 1.85)	− 4.85*	(− 5.20, − 4.50)
65–69	− 2.21*	(− 2.34, − 2.07)	− 4.92*	(− 5.31, − 4.54)
70–74	− 1.99*	(− 2.09, − 1.90)	− 5.19*	(− 5.36, − 5.02)
75–79	− 1.35*	(− 1.44, − 1.27)	− 4.76*	(− 5.04, − 4.48)
80 plus	− 0.48*	(− 0.55, − 0.41)	− 4.27*	(− 4.62, − 3.92)

*ASR*age-standardized rate, *AAPC* average annual percentage changes, *CI* confidence interval

*Significantly different from 0 at alpha = 0.05 (*P* < 0.05)

### Asthma incidence and mortality rates by age, period, and cohort

**Fig. 2:**
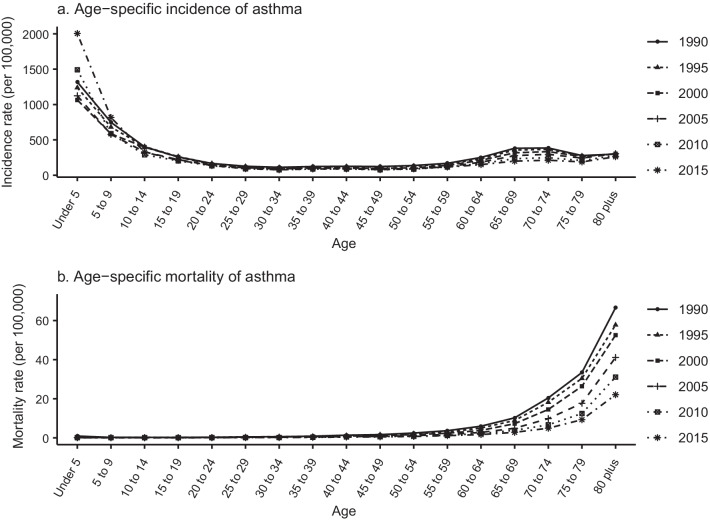
Age-specific asthma incidence (**a**) and mortality (**b**) in Sichuan Province 1990–2019

**Fig. 3:**
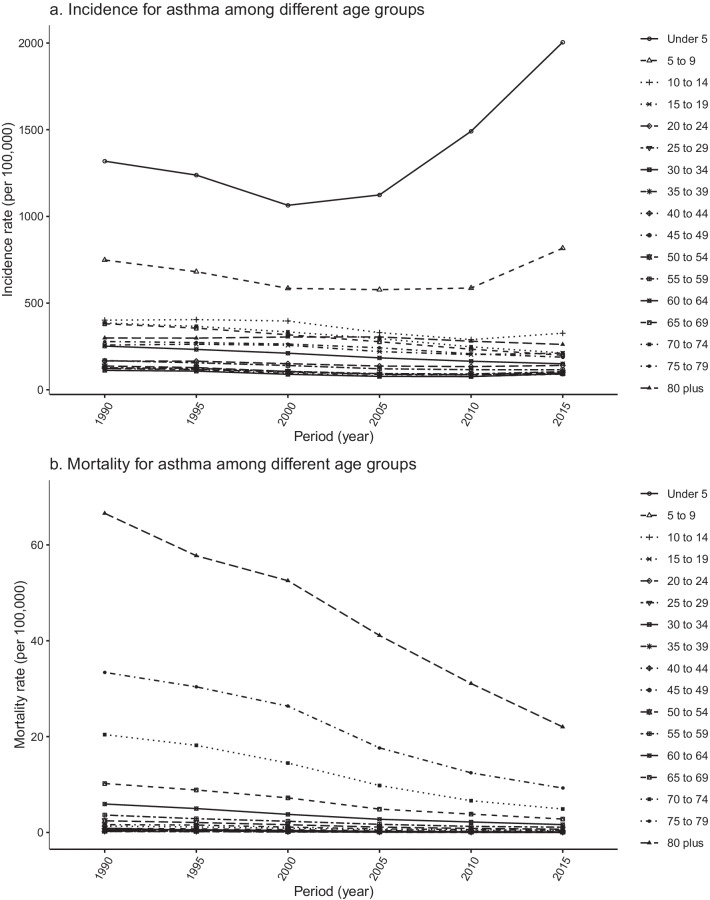
Asthma incidence (**a**) and mortality (**b**) among different age groups in Sichuan Province, 1990–2019

**Fig. 4:**
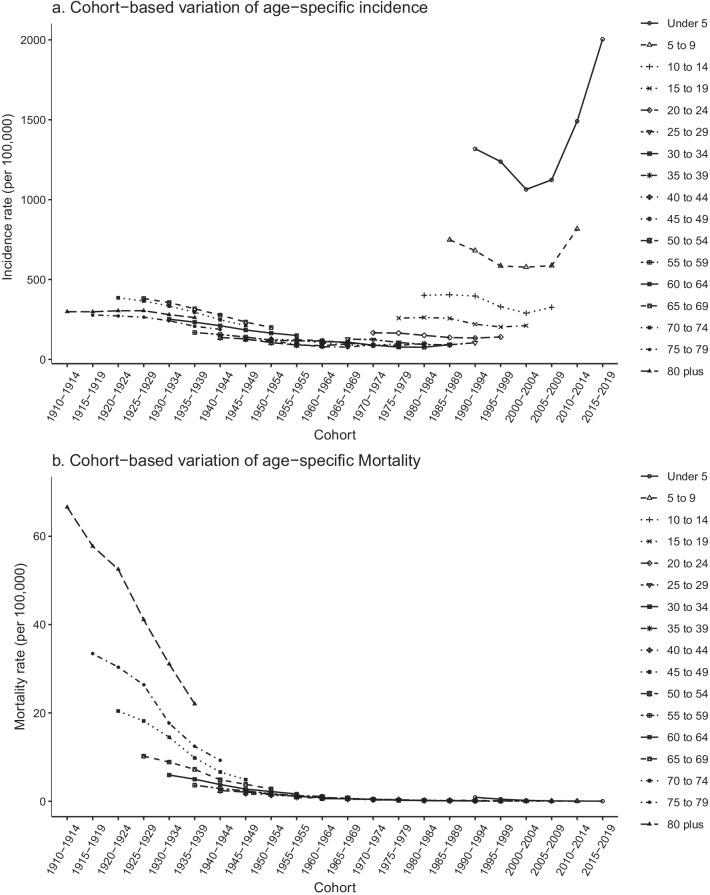
Cohort-based variation in age-specific incidence (**a**) and mortality (**b**) of asthma in Sichuan Province

The trends in age-specific incidence and mortality in 1990–1994, 1995–1999, 2000–2004, 2005–2009, 2010–2014, and 2015–2019 are shown in Fig. 2. The incidence rate was highest in individuals aged < 5 years from 2015 to 2019, and the highest mortality rate was for those aged ≥ 80 years from 1990 to 1994. Figure 3 shows the variation in trends in asthma incidence and mortality for different age groups from 1990 to 2019. The incidence rate of the < 5 years age group and the 5–9 years age group first decreased and then increased over time, whereas it remained almost stable in the other age groups. Mortality in all age groups showed a downward trend between 1990 and 2019. Figure 4 shows the cohort-based variation in age-specific incidence and mortality. The asthma incidence rate in the < 5 years age group and the 5–9 years group first decreased and then increased in later birth cohorts. Mortality in all age groups continued to decline in later birth cohorts.

### Age, period, and cohort effects

**Fig. 5:**
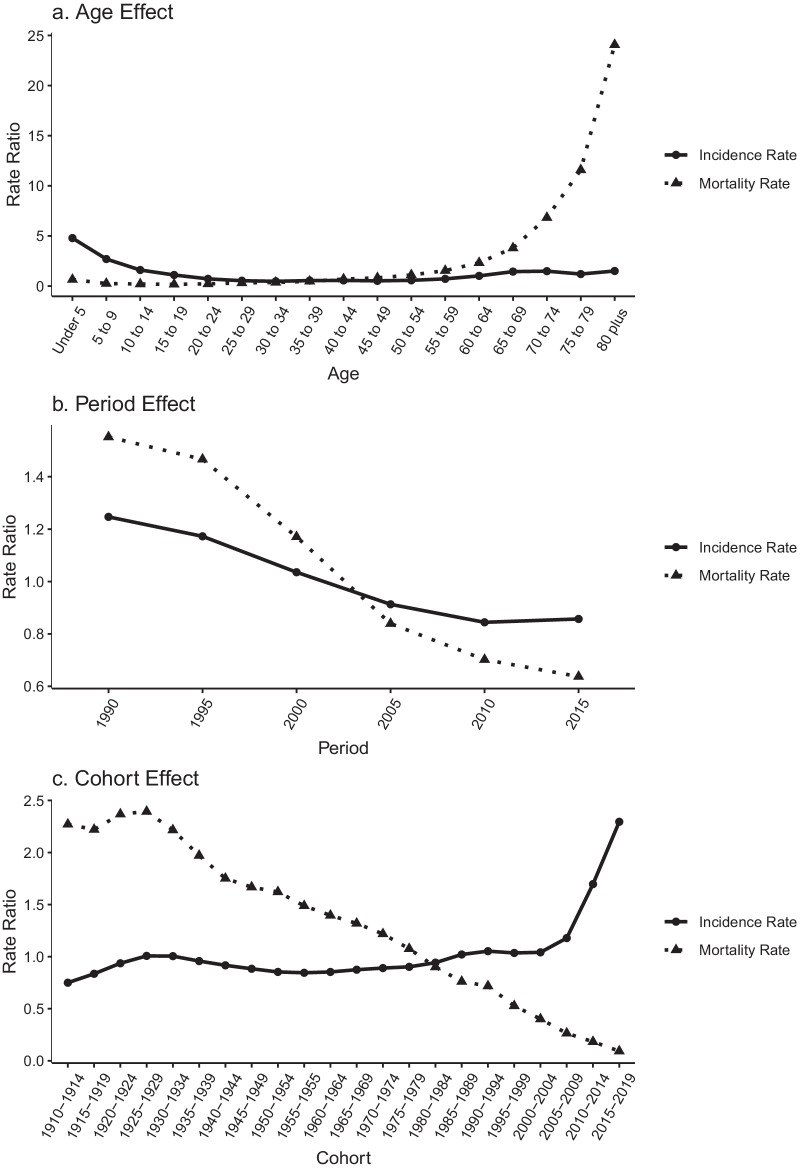
Age (**a**); period (**b**); and cohort (**c**) effects on asthma incidence and mortality rate ratio

Results from the Poisson log-linear model using the intrinsic estimator method are shown in Table 3. The asthma incidence and mortality RR owing to age, period, and cohort effects are shown in Fig. 5.

**Table 3:** Age, period, and cohort effects on asthma incidence and mortality, 1990–2019

Factor	Incidence		Mortality	
	Coefficient	SE	Coefficient	SE
*Age (years)*				
Under 5	1.57*	0.03	− 0.24*	0.02
5–9	0.99*	0.02	− 0.44*	0.06
10–14	0.47*	0.02	− 1.38*	0.05
15–19	0.10*	0.02	− 1.62*	0.05
20–24	− 0.33*	0.02	− 1.76*	0.05
25–29	− 0.63*	0.02	− 1.52*	0.05
30–34	− 0.75*	0.02	− 1.18*	0.05
35–39	− 0.61*	0.02	− 1.03*	0.05
40–44	− 0.58*	0.02	− 0.75*	0.05
45–49	− 0.66*	0.02	− 0.37*	0.05
50–54	− 0.56*	0.02	− 0.20*	0.05
55–59	− 0.34*	0.02	0.10	0.05
60–64	0.01	0.02	0.43*	0.05
65–69	0.36*	0.02	0.84*	0.05
70–74	0.39*	0.02	1.33*	0.05
75–79	0.18*	0.02	1.92*	0.05
80 plus	0.41*	0.03	2.45*	0.05
*Period*				
1990–1994	0.22*	0.01	0.44*	0.03
1995–1999	0.16*	0.01	0.38*	0.03
2000–2004	0.03*	0.01	0.16*	0.03
2005–2009	− 0.09*	0.01	− 0.18*	0.03
2010–2014	− 0.17*	0.01	− 0.35*	0.03
2015–2019	− 0.15*	0.01	− 0.45*	0.03
*Cohort*				
1910–1914	− 0.29*	0.05	0.82*	0.12
1915–1919	− 0.18*	0.04	0.80*	0.09
1920–1924	− 0.07*	0.03	0.86*	0.07
1925–1929	0.01	0.03	0.87*	0.06
1930–1934	0.005	0.03	0.80*	0.06
1935–1939	− 0.04	0.02	0.68*	0.05
1940–1944	− 0.09*	0.02	0.56*	0.05
1945–1949	− 0.12*	0.02	0.51*	0.06
1950–1954	− 0.16*	0.03	0.48*	0.06
1955–1955	− 0.17*	0.03	0.40*	0.06
1960–1964	− 0.16*	0.03	0.33*	0.06
1965–1969	− 0.13*	0.03	0.28*	0.06
1970–1974	− 0.12*	0.03	0.20*	0.06
1975–1979	− 0.1*	0.03	0.07	0.06
1980–1984	− 0.06*	0.02	− 0.10	0.05
1985–1989	0.02	0.02	− 0.27*	0.05
1990–1994	0.05*	0.02	− 0.33*	0.05
1995–1999	0.04	0.02	− 0.64*	0.05
2000–2004	0.04	0.03	− 0.91*	0.06
2005–2009	0.16*	0.03	− 1.33*	0.07
2010–2014	0.53*	0.04	− 1.70*	0.08
2015–2019	0.83*	0.06	− 2.38*	0.13

*SE* standard error

*P < 0.05

After controlling for period and cohort effects, the analysis of the effects of age on asthma showed that the incidence RR was highest in the < 5 years age group, and the mortality RR was highest in the elderly age group (Fig. 5a). The estimated coefficient of the effect of age on incidence for individuals aged < 5 years was 1.57 (*P* < 0.05) and the mortality coefficient for those aged > 80 years was 2.45 (*P* < 0.05), respectively (Table 3). The incidence RR decreased by 3.19 times and the mortality RR increased by 37.33 times in individuals aged > 80 years compared with those aged < 5 years. After controlling for age and cohort effects, the period effect on incidence and mortality decreased from 1990 to 2019 respectively (Fig. 5b). The estimated coefficient for the period effect on incidence and mortality in 2015–2019 was − 0.15 (*P* < 0.05) and − 0.45 (*P* < 0.05), respectively (Table 3). During this period, the incidence and mortality RR of asthma decreased by 1.45 times and 2.44 times, respectively. After controlling for age and period effects, the results demonstrated a cohort effect: the incidence RR increased slowly from the early birth cohorts to the late birth cohorts, particularly after the 2005 birth cohort, whereas the mortality RR continued to decline (Fig. 5c). The estimated coefficient of the cohort effect on incidence and mortality in the 2015–2019 birth cohort was 0.83 (*P* < 0.05) and − 2.38 (*P* < 0.05), respectively (Table 3). Compared with the 1910–1914 birth cohort, the incidence RR and mortality RR of asthma in the 2015–2019 birth cohort increased by 3.06 times and decreased by 24.42 times, respectively.

## Discussion

China’s economy has developed rapidly over the last 40 years. The Great Western Development Strategy was implemented in 2000 to narrow the regional economic gap [37–39]. When the economy is rapidly growing, changes in some diseases may be ignored. There is evidence of an association between asthma and economic development; the Global Asthma Report 2018 indicated that the global asthma prevalence rate has increased by 50% every decade [2, 40]. Currently, there is a lack of systematic reports on asthma in western provinces in China. To address this gap, we described the long-term trends in incidence and mortality rates for asthma in Sichuan Province from 1990 to 2019. Overall, in the latest period of 2015–2019, the highest asthma incidence was 2004.49/100,000 for individuals aged < 5 years, and the highest mortality rate was 22.04/100,000 for those aged > 80 years (Table 1, Fig. 1). The joinpoint regression analysis showed that the asthma ASIR has remained stable from 1990 to 2019. However, the incidence rate has increased in the age groups < 25 years, particularly in the < 5 years age group, and the mortality rate in all age groups has decreased over the last 30 years (Table 2). The APC analysis showed a significant association of age, period, and cohort effects with asthma incidence and mortality in Sichuan Province, during 1990–2019 (Table 3).

The total number of incident asthma cases and ASIR showed abrupt fluctuation from 2015 to 2019, perhaps because the national two-child policy was fully implemented by the end of 2015 [41]. The birth rate in Sichuan has first risen and then fallen since then, with the highest rate in 2017, which may explain the high number of incident asthma cases identified here [42]. Children are more susceptible, which affects the incidence rate. The effects of age, period, and cohort on asthma mortality in Sichuan were not the same as those reported in Huang’s analysis of the long-term trends in asthma mortality in China from 2000 to 2019 [14]. Studies in Sichuan and China as a whole have shown that the age effect on asthma mortality increases with age, and the risk of mortality in later birth cohorts has generally decreased; however, the national period effect showed a V-shaped trend, which gradually decreased by period in Sichuan.

The results showed that age had a significant and positive effect on asthma incidence in the age groups < 20 years (particularly the group < 5 years) and > 65 years, whereas asthma mortality remarkably increased with advancing age, mainly in elderly individuals (Fig. 5a). Asthma is a complex disease that often starts in childhood [43], and is sometimes the main cause of childhood disease burden in low- and low-middle social-demographic index countries [44, 45]. At present, the global incidence rate of asthma in younger individuals is decreasing, whereas it is increasing in Sichuan (Figs. 2, 3) [2]. If childhood asthma is not effectively controlled, it can lead to sustained, large economic and health burdens for families and society and a range of negative effects that may continue into adulthood, such as problems with children’s growth, development, and learning, and an increased risk of chronic obstructive pulmonary disease [12, 46–48]. There are many influencing factors of childhood asthma that may increase its incidence, including virus infection, air pollution, genetic susceptibility, obesity, population aggregation, and abnormal immune maturation in early life.[43]Changes in lifestyle and environmental exposures, rather than population genetics, may be the main causes of the rise in asthma in children. It has been reported that asthma incidence rates have increased rapidly in cities in low- and middle-income countries, which suggests that urbanization may increase asthma risk factors or inhibit protective factors, or both [49]. Childhood obesity is another important asthma risk factor; the childhood obesity rate in China has been increasing in recent years. From 1991 to 2015, the prevalence of obesity among Chinese children and adolescents showed an upward trend that was higher in urban areas than in rural areas, in boys than in girls, and in infancy than in other growth stages [50]. Therefore, public health measures to reduce childhood asthma are needed, such as integrating childhood asthma diagnosis into primary healthcare for children and adolescents, providing pregnant women with vitamin D or fish oil supplements (or both), controlling childhood obesity, and providing protection for children by regulating immune function [43, 51–53]. Elderly people in Sichuan have the highest asthma mortality and a high chronic respiratory disease mortality [54]. Generally, there is a greater cumulative effect of risk factors and a greater probability of the coexistence of multiple diseases, which may increase asthma mortality in elderly people [55]. Important risk factors that increase the incidence or mortality of adult asthma include smoking, occupational exposure, air pollution, cold weather, and comorbid disease [52]. It is likely that public policies such as smoking bans in public places and strengthening occupational protection measures will play an important role in reducing the incidence and mortality of asthma in China’s rapidly aging population.

The period effect is usually affected by a series of complex historical events and environmental factors, such as wars, infectious disease epidemics, public health interventions, and socioeconomic development [17]. This study showed a decreasing trend for the effect of period on overall asthma incidence and mortality in Sichuan Province from 1990 to 2019 (Fig. 5b). Following global progress in asthma pathogenesis and treatment, asthma has become a controllable chronic disease [56–61]. This decreasing trend may reflect better access to health services and improved treatments [62]. The GBD study 2019 Universal Health Coverage Collaborators reported the effective medical service coverage level worldwide. Healthcare coverage ensures that all people can access the health services they need without financial difficulties. China has significantly improved its medical service level, and has an effective universal health coverage index rating of 70 points and an asthma treatment service index rating of 86 points, which indicates medium and high service quality levels, respectively [63].

The cohort effect represents variations between groups of individuals born in the same year, which could arise if each cohort has accumulated a different burden of physical and social exposures from gestation to old age [17]. Figure 5 showed that the RR of the cohort effect in asthma mortality has decreased, whereas the RR of asthma incidence in later birth cohorts has increased. Combined with the increase in asthma incidence rate in the younger age groups from 2000 (Fig. 3) and the greater asthma incidence in the younger age groups in later birth cohorts (Fig. 4), these findings suggest that later birth cohorts may experience or accumulate more asthma risk factors in their lifetime. This may indicate that the rapid economic development and accelerated urbanization in Sichuan Province over the last 20 years is related to the increased incidence rate of childhood asthma. Some studies have suggested that increasing economic development and rapid urbanization increase asthma risk factors (such as allergens and irritants), thus increasing asthma prevalence [64]. The rapid and sudden increase in asthma incidence and prevalence in Western developed countries may be related to rapid economic development [65, 66]. In addition, the high smoking rate in China may partly explain the cohort effect of asthma. Smoking rates are high among Chinese teenagers and adults, and active and passive smoking has increased the incidence of asthma in adults and non-smokers [67–69]. Wang and his colleagues collected data from a series of cross-sectional surveys conducted in China of national health services from 2003 to 2013. The results showed that smoking rates of male and female adolescents have substantially increased, and the smoking rate of young women has steadily increased. China remains the largest tobacco consumer in the world, with a large burden of chronic diseases related to smoking [70].

To summarize, the present findings indicate that asthma incidence and mortality in children and elderly people in Sichuan Province over the last 30 years warrants more attention. In 2020, the Chinese government proposed to strength the Great Western Development Strategy [71]. Given population aging and the implementation of the multi-child policy, positive and effective measures are needed in Sichuan Province to reduce the incidence and mortality of asthma.

To our knowledge, this is the first study to provide information on changes in asthma incidence and mortality in western province of China over the last 30 years. The findings of this APC effect analysis of asthma morbidity and mortality provide epidemiological data that may increase understanding of the causes of the increased asthma burden. However, our study had several limitations. First, we studied long-term changes by age in asthma in Sichuan. However, owing to insufficient data, we were unable to estimate the incidence and mortality trends in urban and rural areas in Sichuan. Second, although the GBD study used many methods to reduce data bias, including error classification correction, addressing incompleteness, and redistribution of “garbage” code, it may be difficult to completely avoid data inaccuracy. Therefore, our results should be treated with caution. Third, as there was no specific analysis of the relationship between asthma risk factors and morbidity and mortality, the reasons for the continuous upward trend in the cohort effect on childhood asthma remain to be clarified.

## Conclusion

In this study, we identified significant age, period, and cohort effects of asthma incidence and mortality in Sichuan Province from 1990 to 2019. There was a significant effect of older age on the asthma mortality rate over the last 30 years, and the incidence rate in children aged < 5 years increased. The relative risk of asthma incidence in later birth cohorts increased. With economic improvements and the rapid urbanization in western China, effective preventive measures and public health policies should be implemented to protect children and elderly people from potentially harmful chronic diseases.

## Data Availability

National data can be accessed from the following link in public: For the GBD results see http://ghdx.healthdata.org/gbd-results-tool. For the online visualization tools see https://vizhub.healthdata.org/gbd-compare. For the GBD Data Resources see http://ghdx.healthdata.org/gbd-2019. Provincial data were not publicly available, but can be requested through contact with the corresponding authors.

## Abbreviations

APC: Age-period-cohort
ASIR: Age-standardized incidence rate
ASMR: Age-standardized mortality rate
AAPC: Average annual percent change
DALY: Disability adjusted life years
GBD: Global Burden of Disease Study
RR: Rate ratio
